# Review of the In Vitro Microbiological Activity of Mecillinam Against Common Uropathogens in Uncomplicated Urinary Tract Infection: Focus on Resistant Pathogens

**DOI:** 10.1093/ofid/ofae296

**Published:** 2024-05-24

**Authors:** Thomas P Lodise, Keith S Kaye, Anne Santerre Henriksen, Gunnar Kahlmeter

**Affiliations:** Department of Pharmacy Practice, Albany College of Pharmacy and Health Sciences, Albany, New York, USA; Department of Medicine, Rutgers Robert Wood Johnson Medical School, New Brunswick, New Jersey, USA; Maxel Consulting ApS, Jyllinge, Denmark; Department of Clinical Microbiology, Central Hospital, Växjö, Sweden

**Keywords:** Enterobacterales, ESBL, mecillinam, resistance, uncomplicated urinary tract infection

## Abstract

Antimicrobial resistance in uropathogens commonly causing urinary tract infections (UTIs) is a growing problem internationally. Pivmecillinam, the oral prodrug of mecillinam, has been used for over 40 years, primarily in Northern Europe and Canada. It is recommended in several countries as a first-line agent for the treatment of uncomplicated UTIs (uUTIs) and is now approved in the United States. We performed a structured literature search to review the available evidence on susceptibility of common uUTI-causing uropathogens to mecillinam. Among 38 studies included in this literature review, susceptibility rates for *Escherichia coli* to mecillinam—including resistant phenotypes such as extended-spectrum β-lactamase–producing *E. coli*—exceed 90% in most studies. High rates of susceptibility were also reported among many other uropathogens including *Klebsiella* spp., *Enterobacter* spp., and *Citrobacter* spp. In the current prescribing climate within the United States, pivmecillinam represents a viable first-line treatment option for patients with uUTI.

## Teaser

Pivmecillinam, used for >40 years to treat uncomplicated urinary tract infections, is supported by a wealth of microbiological data. Common uropathogens are highly susceptible to mecillinam at current susceptibility breakpoints, also in the face of the most common β-lactam resistance mechanisms.

Urinary tract infections (UTIs) are the most common bacterial infection in the United States and elsewhere, and *Escherichia coli* is the most common pathogen [1]. Although UTIs can be associated with serious illness [2], the majority of cases are considered uncomplicated UTI (uUTI) [3]. These predominantly affect adult women, in whom lifetime incidence is 50%–60% [1]. Aside from a peak in young women in their teens and early 20s (around one-fifth of whom experience a UTI annually [4]), the prevalence of UTIs increases with age [1]. It has been estimated that 150 million UTIs occur worldwide each year and that the associated societal costs (in 2015, including health care costs and time missed from work) in the United States alone are ∼$3.5 billion per year [2].

One major challenge associated with the management of uUTIs is the increasing proportion of infections caused by antibiotic-resistant gram-negative bacteria [5, 6]. It is estimated that one-fifth of adult outpatients with a uUTI will receive empiric treatment with an antibiotic to which the causative uropathogen is resistant [7]. Resistance to both fluoroquinolones and trimethoprim-sulfamethoxazole (TMP-SMX) among *E. coli* has been reported to exceed 20% in most regions of the United States [7–9]. The prevalence of extended-spectrum β-lactamase–producing Enterobacterales (ESBL Enterobacterales) in the community setting is also on the rise. In the United States, the percentage of isolates with an ESBL-producing phenotype increased by 30% between 2011 and 2020 (from 6.5% to 9.4%) [10]. Consequently, there is an urgent need to ensure prudent use of currently available antimicrobials, including those with low resistance profiles.

Pivmecillinam, an oral prodrug of mecillinam, has been approved for the treatment of uUTI in Canada and Europe since the 1980s [11–13]. It is recommended as a first-line agent for, the management of uUTIs in several expert guidelines including those from the Infectious Diseases Society of America (IDSA) and the European Society of Clinical Microbiology and Infectious Diseases [3, 14, 15]. The efficacy and safety of pivmecillinam have been comprehensively documented over the years. Microbiological response rates for pivmecillinam in uUTI have been reported to be between 75% and 94% [16–19], with clinical response rates ranging from 82% to 95% [17, 19, 20]. A meta-analysis examining efficacy and safety data from 24 randomized controlled trials published between 1977 and 2009 concluded that the pivmecillinam regimens administered in the reviewed trials were too diverse to make any firm recommendations around dose, frequency of dose, or treatment duration and that all regimens had similar efficacy and safety [21].

Pivmecillinam has been used for decades in Denmark, Norway, and Sweden as the principal antibiotic for uUTI treatment; not only has it demonstrated clinical effectiveness, but resistance rates have also remained consistently low (4%–6%) over >40 years of use [22]. Despite this extensive clinical history of use in some countries, pivmecillinam has only recently (2024) received approval in the United States for the treatment of uUTIs by the US Food and Drug Administration (FDA) [23]. It is timely to review the evidence of the susceptibility of common uUTI-causing uropathogens to mecillinam and describe the available susceptibility surveillance data.

## LITERATURE SEARCH METHODOLOGY

A structured literature search was conducted to identify available data on mecillinam/pivmecillinam microbiological activity against Enterobacterales. As pivmecillinam is an oral prodrug, antibiotic susceptibility testing (AST) is performed against mecillinam, and both pivmecillinam and mecillinam were captured in the literature review. The focus was to identify mecillinam/pivmecillinam susceptibility data against Enterobacterales isolates including urinary isolates from individuals with uUTI. As part of the literature review, we assessed whether there were any changes in the resistance profile of pivmecillinam over time among any studies that evaluated temporal trends in susceptibility.

A search was performed via PubMed on November 30, 2023, using the following terms: ((pivmecillinam) OR (mecillinam)) AND (urinary tract infection) AND ((susceptibility) OR (resistance) OR (efficacy)) AND ((ESBL) OR (lactamase) OR (Enterobacteriaceae) OR (Enterobacterales)) AND (English [Language]). No time limits were imposed on publication date to review any evidence of evolution of resistance over time. Only articles with abstracts available were considered (n = 112). Articles were reviewed at the abstract level for evidence of data on susceptibility/resistance rates of pivmecillinam/mecillinam in uUTI; those considered to provide representative data from adequately sized samples (≥10 isolates) were included, and duplicated data were excluded. Data relating to gram-positive bacteria (such as *Staphylococcus saprophyticus* and *Enterococcus*) and non-Enterobacterales gram-negative bacteria were excluded. Studies specifically conducted in men or pediatric patients were excluded. However, those without specific demographic criteria or with mixed populations including men or children were included. Publications cited by identified articles and any additional relevant published material not identified from the initial searches were included at the authors' discretion but were subjected to the same inclusion/exclusion criteria on screening.

Included studies were further limited to only those that apply breakpoints published by the European Committee on Antimicrobial Susceptibility Testing (EUCAST) or Clinical and Laboratory Standards Institute (CLSI) [24–26]. When analyzing AST data from studies using different susceptibility breakpoints, it is important to note that both EUCAST and CLSI identify 8 mg/L as the susceptibility breakpoint for uUTI [24]. Agar dilution is the reference method for mecillinam minimal inhibitory concentration (MIC) determination [25, 27]. On this basis, only studies using appropriate AST methodology were included in the review (eg, agar dilution or standardized and quality-controlled disk diffusion). Studies using broth microdilution were excluded as this methodology is not recommended for determination of mecillinam susceptibility [27]. Some of the studies performed before 2005 referred to nationally defined breakpoints (eg, Comité de l′ Antibiogramme de la Société Française de Microbiologie [CA-SFM] in France; Swedish Reference Group for Antibiotics [SRGA] in Sweden), but they were included because they used the EUCAST methodology.

### Susceptibility of uUTI-Causing Uropathogens to Mecillinam

**Figure 1.**
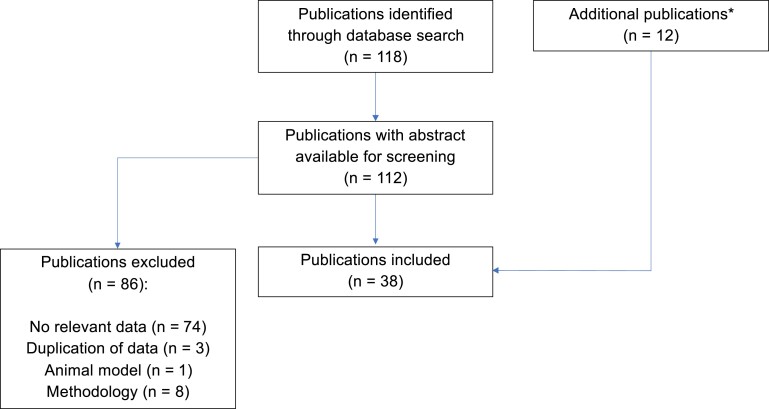
Identification of literature for mecillinam susceptibility testing. *Identified through bibliographies of included publications or known to authors and included 2 publications that had previously been identified by the database search but had been excluded due to lack of abstract for screening.

The identification of literature via the structured search is summarized in Figure 1.

A comprehensive summary of retrieved data is tabulated across Tables 1 and 2. In Table 1, all studies with published susceptibility data for *E. coli* against mecillinam are described, divided into 3 sections—overall, ESBL-producing phenotype, and carbapenem-resistant. Similarly, in Table 2, published susceptibility data for non–*E. coli* Enterobacterales against mecillinam are summarized, divided into the following 3 sections: overall, ESBL- or AmpC-producing phenotype, and carbapenem-resistant. The following text describes key themes arising from our review of these data.

**Table 1.** Published Susceptibility Data for *E. Coli* Against Mecillinam: Overall and Resistant Phenotypes

Study (Period Studied)	Origin of Isolates	Uropathogen	No. of Isolates	Percent Susceptibility (AST Method Used)	MIC Information, mg/L
*E. coli*: overall					*…*
Frimodt-Møller et al., 2023 [22] (2010 to 2020)	Denmark	*E. coli*	649 639^a^	94.5 (disk diffusion)	Not reported
	Norway	*E. coli*	15 018^a^	94.9	
	Sweden	*E. coli*	977 400^a^	95.7	
Farfour et al., 2022 [28] (September 1, 2017, to August 31, 2018)	France	*E. coli*	96 597	91.4 (disk diffusion)	Not reported
Tutone et al., 2022 [29] (April 2019 to November 2019)	Belgium, UK, Italy, Spain, and Russia	*E. coli*	2061	91.8 (disk diffusion)	Not reported
Kresken et al., 2022 [30] (October 2019 to March 2020)	Germany	*E. coli*	414	95.2 (agar dilution)	MIC_50_ 0.5 MIC_90_ 4 MIC range 0.06 to >32
Olenski et al., 2021 [31] (July 1, 2018, to December 31, 2019)	Australia	*E. coli* susceptible to third-generation cephalosporin	101	83.2 (disk diffusion)	Not reported
Thaulow et al., 2021 [32] (2013 to 2017)	Norway	*E. coli* from children	403	96.0 (disk diffusion)	Not reported
		*E. coli* from adults	6105	94.3	
Boel et al., 2019 [33] (January 1, 2010 to September 30, 2016)	Denmark	*E. coli*	4837	3-d course: 97.8 (disk diffusion)	Not reported
			9411	5-d course: 97.3	
			7616	7-d course: 96.8	
Jansåker et al., 2019 [34] (January 1, 2010, to September 30, 2016)	Denmark	Non-ESBL *E. coli*	36 186	97.5 (disk diffusion)	Not reported
Ny et al., 2019 [35] (October 2015 to January 2017)	Finland, Latvia, Poland, Russia, and Sweden	*E. coli*	775	95.9 (disk diffusion)	Not reported
Duployez et al., 2016 [36] (March 15 to July 4, 2015)	France	*E. coli* from pregnant women	235	86.4 (disk diffusion)	Not reported
Kahlmeter et al., 2015 [37] (2000, 2008, and 2014)	France	*E. coli*	166	97.0 (disk diffusion)	Not reported
	Germany	*E. coli*	133	97.0	
	Spain	*E. coli*	169	93.5	
	Sweden	*E. coli*	137	98.5	
	UK	*E. coli*	124	95.2	
Giske et al., 2015 [38] (2011 to 2013)	Sweden	*E. coli*	22 142–23 951 per year	95–96 (disk diffusion)	Not reported
Etienne et al., 2014 [39] (2009 to 2011)	France	*E. coli*	157	87 (disk diffusion)	Not reported
Sundvall et al., 2014 [40] (January to March of 2003 and 2012)	Sweden	*E. coli*	260	94.2 (disk diffusion)	Not reported
Kahlmeter et al., 2012 [41] (2007 to 2008)	Austria	*E. coli*	146	100 (disk diffusion)	Not reported
	Greece	*E. coli*	209	98.6	
	Portugal	*E. coli*	144	98.6	
	Sweden	*E. coli*	203	99.5	
	UK	*E. coli*	201	99.0	
Lampri et al., 2012 [42] (January 2005 to March 2006)	Greece	*E. coli*	1606	95.8 (agar dilution)	Not reported
Lindbäck et al., 2010 [43] (January to February 2008)	Sweden	*E. coli*	205	98.7 (disk diffusion)	Not reported
Schito et al., 2009 [44] (September 2003 to June 2006)	Europe and Brazil	*E. coli*	2313	95.8 (and 1.5 intermediate; agar dilution)	MIC_50_ 0.25 MIC_90_ 2 MIC range <0.12 to >128
Issack et al., 2007 [45] (January 15 to April 15, 2005)	Mauritius	*E. coli*	121	82.6 (and 14.0 intermediate; disk diffusion)	Not reported
Abelson Storby et al., 2004 [46] (1990 to 2001)	Sweden	*E. coli*	Not reported	>98 in 2001 (disk diffusion)	Not reported
Kahlmeter et al., 2003 [47] (January 1999 to December 2000)	Europe and Canada	*E. coli*	2478	98.8 (disk diffusion)	Not reported
Mazzulli et al., 2001 [48] (July to December 1997)	Canada	*E. coli*	1832	99.7 (agar dilution)	Not reported
*E. coli*: ESBL-producing phenotype					*…*
Plambeck et al., 2022 [49] (March 2019 to January 2020)	Germany	ESBL *E. coli*	177	97.0 (agar dilution and disk diffusion)	MIC_50_ 2 MIC range 0.25 to 64
		AmpC *E. coli*	11	100	MIC_50_ 1 MIC range 0.5 to 4
Tutone et al., 2022 [29] (April to November 2019)	Belgium, UK, Italy, Spain, and Russia	Cefpodoxime-resistant *E. coli*	348	82.5 (disk diffusion)	Not reported
Kresken et al., 2022 [30] (October 2019 to March 2020)	Germany	ESBL *E. coli*	46	91.3 (agar dilution)	MIC_50_ 1 MIC_90_ 4 MIC range 0.25 to >32
Olenski et al., 2021 [31] (July 1, 2018, to December 31, 2019)	Australia	*E. coli* nonsusceptible to third-generation cephalosporin	98	87.8 (disk diffusion)	Not reported
Fuchs et al., 2019 [50] (November 2016 to March 2017)	Germany	ESBL *E. coli*	86	96.5 (disk diffusion)	MIC_50_ 0.5 MIC_90_ 1
Jansåker et al., 2019 [34] (January 1, 2010, to September 30, 2016)	Denmark	ESBL *E. coli*	1619	89.3 (disk diffusion)	Not reported
Priyadharshana et al., 2019 [51] (December 2016 to March 2017)	Sri Lanka	ESBL *E. coli*	68	91 (and 6% intermediate; disk diffusion)	Not reported
Raja, 2019 [52] (September 2015 to September 2017)	UK	ESBL *E. coli*	889	96 (disk diffusion)	Not reported
Bouxom et al., 2018 [53] (throughout 2016)	France	ESBL *E. coli*	100	92 (agar dilution)	MIC_50_ 1 MIC_90_ 8 MIC range 0.06 to >128
Zykov et al., 2016 [54] (2010 to 2011)	Norway	ESBL *E. coli*	105	94 (disk diffusion)	MIC_50_ 1 MIC_90_ 4 MIC range 0.064 to 256
Giske 2015 [38] (2011 to 2013)	Sweden	ESBL *E. coli*	637–830	∼94–95 (disk diffusion)	Not reported
Fournier et al., 2013 [55] (June 2009 to September 2010)	France	ESBL *E. coli*	100	90 (agar dilution)	MIC_50_ 1 MIC_90_ 8 MIC range 0.25 to >128
Lampri et al., 2012 [42] (January 2005 to March 2006)	Greece	ESBL *E. coli*	48	97.9 (agar dilution)	MIC_50_ 1 MIC_90_ 4 MIC range 0.25 to 16
Titelman et al., 2011 [56] (2005)	Sweden	ESBL *E. coli*	149	87 (disk diffusion)	MIC range 0.064 to >256
Tärnberg et al., 2011 [57] (January 2002 to December 2007)	Sweden	CTX-M-producing *E. coli*	198	91.4 (disk diffusion)	MIC_50_ 1 MIC_90_ 8 MIC range 0.125 to >128
*E. coli*: carbapenem-resistant					*…*
Emeraud et al., 2022 [58] (January 2019 to June 2021)	France	Carbapenem-resistant *E. coli* (all mechanisms)	1943	84.2 (disk diffusion)	Not reported
		ESBL/AmpC *E. coli*	276	75	
		OXA-48-like *E. coli*	1198	92.6	
		NDM *E. coli*	391	76.2	
		VIM *E. coli*	34	17.6	
		KPC *E. coli*	17	0	
Tsakris et al., 2022 [59] (2012 to 2019)	Greece	OXA-48-like-producing Enterobacterales	17	94.1 (MIC gradient strips on agar)	MIC range 0.5 to 32
Fuchs et al., 2021 [60] (not reported)	Germany	Carbapenemase-producing Enterobacterales	30	40 (agar dilution and disk diffusion)	MIC_50_ 32 MIC_90_ >256 MIC range 1 to >256
Mutters et al., 2015 [61] (not reported)	Germany	*E. coli* with decreased susceptibility to carbapenem	20	65 (MIC gradient strips on agar)	MIC_50_ 6 MIC_90_ 256 MIC range 1 to >256

^a^Numbers represent 10 years of data, where yearly numbers for 2010–2020 have been added.

Abbreviations: AST, antibiotic susceptibility test; ESBL, extended-spectrum β-lactamase; MIC, minimal inhibitory concentration; UK, United Kingdom.

**Table 2.** Published Susceptibility Data for Non–*E. coli* Enterobacterales (Includes Unspecified Bacteria) Against Mecillinam: General and Resistant Phenotypes

Study (Period Studied)	Origin of Isolates	Uropathogen	No. of Isolates	Percent Susceptibility (AST Method Used)	MIC Information, mg/L
Non–*E. coli* (includes unspecified) Enterobacterales: overall					
Farfour et al., 2022 [28] (September 1, 2017, to August 31, 2018)	France	*K. pneumoniae* complex	13 014	88.7 (disk diffusion)	Not reported
		*P. mirabilis*	7781	73.2	
		*Enterobacter cloacae* complex	3891	88.4	
		*C. koseri*	3086	97.9	
		*K. oxytoca*	2549	89.2	
		*M. morganii*	2012	29.5	
		*K. aerogenes*	1744	85.3	
		*C. freundii*	1342	87.4	
		*P. vulgaris*	805	68.2	
		*S. marcescens*	402	30.8	
Issack et al., 2007 [45] (January 15 to April 15, 2005)	Mauritius	*Klebsiella* spp.	36	80.6 (and 16.7 intermediate; disk diffusion)	Not reported
		*Proteus* spp. and other Enterobacterales	55	85.5 (and 12.7 intermediate)	
Kahlmeter et al., 2003 [47] (January 1999 to December 2000)	Europe and Canada	*P. mirabilis*	192	95.8 (disk diffusion)	Not reported
		*Klebsiella* spp.	97	94.8	
		Other Enterobacterales	122	98.4	
Mazzulli et al., 2001 [48] (July to December 1997)	Canada	*Klebsiella* spp.	78	100 (agar dilution)	Not reported
		*Enterobacter* spp.	19	100	
		*Proteus* spp.	41	97.6	
		*Citrobacter* spp.	14	92.8	
Anderson et al., 1976 [62] (not reported)	UK	Enterobacterales	705	General practice/antenatal: 99.6 (disk diffusion)	Not reported
			227	Hospital outpatients: 99.6	
			1068	Hospital inpatients: 96.9	
Non–*E. coli* (includes unspecified) Enterobacterales: ESBL- or AmpC-producing phenotypes					
Plambeck et al., 2022 [49] (March 2019 to January 2020)	Germany	MDR *Klebsiella* spp.	87	69.0 (agar dilution and disk diffusion)	MIC_50_ 8 MIC range 0.5 to >128
		MDR *K. pneumoniae* (60/66 ESBL)	66	76.0	MIC_50_ 4 MIC range 0.5 to >128
		MDR *Enterobacter* spp. (50/52 AmpC)	52	96.0	MIC_50_ 1 MIC range 0.125 to >128
		MDR *C. freundii* (24/33 AmpC)	33	73.0	MIC_50_ 2 MIC range 0.125 to >128
		MDR *M. morganii* (10/11 AmpC)	11	0.0	MIC_50_ >128 MIC range 128 to >128
Fuchs et al., 2019 [50] (November 2016 to March 2017)	Germany	MDR *Klebsiella* spp.	18	88.9 (disk diffusion)	MIC_50_ 0.5 MIC_90_ 6
Raja, 2019 [52] (September 2015 to September 2017)	UK	ESBL Enterobacterales	986	95 (disk diffusion)	Not reported
		ESBL *Klebsiella* spp.	71	83	
Bouxom et al., 2018 [53] (throughout 2016)	France	ESBL *K. pneumoniae*	50	90 (agar dilution)	MIC_50_ 2 MIC_90_ 8 MIC range 0.5 to >128
O’Kelly et al., 2016 [63] (2012 to 2013)	UK	ESBL Enterobacterales	95	94.74 (MIC gradient)	MIC range 0.25 to 256
Deshpande et al., 2013 [64] (not reported)	UK	ESBL Enterobacterales	336	87.8 (disk diffusion)	Not reported
Non–*E. coli* (includes unspecified) carbapenem-resistant Enterobacterales					**…**
Plambeck et al., 2022 [49] (March 2019 to January 2020)	Germany	OXA-48-like (*C. freundii*, *E. coli*, and *K. pneumoniae*)	8	50.0 (agar dilution and disk diffusion)	MIC_50_ 8 MIC_90_ 64 MIC range 1 to 128
		VIM/NDM (*P. mirabilis* and *C. freundii*)	3	0.0	MIC_50_ >128 MIC_90_ >128 MIC range >128
		KPC (*C. freundii*)	1	0.0	MIC range >128
Emeraud et al., 2022 [58] (January 2019 to June 2021)	France	Carbapenem-resistant *K. pneumoniae*	2511	66.9 (disk diffusion)	Not reported
		Carbapenem-resistant *E. cloacae* complex	1775	75	
		Carbapenem-resistant *Citrobacter* spp.	1295	64.7	
		Carbapenem-resistant *K. oxytoca*	262	60.7	
		Carbapenem-resistant *K. aerogenes*	257	64.6	
		Carbapenem-resistant *Serratia* spp.	86	33.7	
		Carbapenem-resistant *M. morganii*	43	11.6	
		Carbapenem-resistant *Proteus* spp.	46	84.8	
Fuchs et al., 2021 [60] (not reported)	Germany	Carbapenemase-producing Enterobacterales: *K. pneumoniae*	47	8.5 (agar dilution and disk diffusion)	MIC_50_ 256 MIC_90_ >256 MIC range 4 to >256
		Carbapenemase-producing Enterobacterales: *E. cloacae*	13	53.8	MIC_50_ 8 MIC_90_ >256 MIC range 2 to >256
Tsakris et al., 2022 [59] (2012 to 2019)	Greece	OXA-48-like carbapenemase-producing Enterobacterales	180	94.4 (MIC gradient strips on agar)	MIC range 0.5 to >256
Mutters et al., 2015 [61] (not reported)	Germany	*K. pneumoniae* with decreased susceptibility to carbapenem	20	30 (MIC gradient strips on agar)	MIC_50_ 256 MIC_90_ 256
		*E. cloacae* with decreased susceptibility to carbapenem	20	80	MIC_50_ 3 MIC_90_ 256
		*Enterobacter aerogenes* with decreased susceptibility to carbapenem	17	65	MIC_50_ 6 MIC_90_ 256

Abbreviations: AST, antibiotic susceptibility test; ESBL, extended-spectrum β-lactamase; MDR, multidrug-resistant; MIC, minimal inhibitory concentration.

### Susceptibility of *E. coli* (Overall) to Mecillinam

Twenty-two studies reported data on the overall susceptibility of mecillinam for *E. coli* (Table 1) [22, 28–48]. In most of these studies, the susceptibility to mecillinam exceeded 95%. Only 4 reported susceptibilities of <90% (and all were >80%). The largest study sample (>1 500 000 isolates studied over a decade), and one of the most recent, was examined in an analysis of mecillinam resistance rates in *E. coli* gathered between 2010 and 2020 from the monitoring programs of Denmark, Norway, and Sweden [22]. Susceptibility to mecillinam remained stable over the 11-year period between 94% and 96%. The authors also extracted consumption data and analyzed the association between consumption and resistance; interestingly, consumption of pivmecillinam increased significantly over time in Denmark with no attendant rise in resistance—in fact, resistance rates significantly decreased [22]. A multinational, prospective, multicenter study (ECO-SENS) investigated the prevalence and antimicrobial susceptibility of community-acquired uropathogens at long-range intervals in women aged 18–65 years with symptoms of uncomplicated lower UTI [47]. A total of 2478 *E. coli* isolates from women with uUTI in primary care in 17 countries were examined from 1999 to 2000. All susceptibility tests were performed in 1 laboratory, and overall resistance to mecillinam was 1.2% [47]. In a follow-up ECO-SENS study in 2008, resistance to mecillinam in 903 *E. coli* isolates from women with community-acquired UTIs in Austria, Greece, Portugal, Sweden, and the United Kingdom ranged from 0% to 1.4% (0.9% overall) [41]. In a 2015 publication of ECO-SENS data, Kahlmeter et al. reported that susceptibility of *E. coli* to mecillinam remained above 95% between 2000 and 2014 in France, Germany, Sweden, and the United Kingdom. In Spain, the value dropped from 99.0% in 2000 to 93.5% in 2014 [37]. Slightly lower susceptibility of *E. coli* to mecillinam was reported in a 2022 cross-sectional study of samples from nonhospitalized women (91.8%) across Europe, with the highest susceptibility reported in Russia (94.8%) and the lowest in Italy (89.2%) [29].

Data from a large university laboratory in Sweden were reported in a review article by Giske et al. in 2015 [38]. These data showed that overall resistance in *E. coli* for the years 2011 to 2013 (based on >20 000 tested isolates per year) was between 4% and 5%. A study investigating resistance to mecillinam in *E. coli* isolates from urine specimens of adult primary care patients in Germany (86.0% of samples from female patients) in 2019 and 2020 reported that 394 of 414 (95.2%) non-ESBL isolates were inhibited by mecillinam [30].

### Susceptibility of ESBL-Producing Phenotypes of *E. coli* to Mecillinam

Nineteen publications were identified as reporting data on the susceptibility of resistant phenotypes of *E. coli* to mecillinam (Table 1) [29–31, 34, 38, 42, 49–61]. Four of these studies reported susceptibility data relating to carbapenem-resistant *E. coli* [58–61].

Among the 15 studies reporting data on resistant phenotypes of *E. coli* excluding carbapenem-resistant strains, mainly ESBL-producing *E. coli*, most reported susceptibility to mecillinam of >90%, and all reported rates of at least 82.5%. The largest sample of isolates was reported in a Danish retrospective cohort study comprising men and women with significant bacteriuria for *E. coli* (≥10^3^ colony-forming units/mL) to whom a simultaneous oral empirical antibiotic for UTI had been given [34]. A total of 1619 cases of community-acquired UTIs attributable to ESBL-producing *E. coli* were identified, and resistance to mecillinam was reported to be 10.7% [34]. In a retrospective study of data from 2015 to 2017 in the United Kingdom using samples from patients from the community and hospital with confirmed UTIs due to ESBL-producing Enterobacterales, mecillinam sensitivity was observed in 96% (855/889) of ESBL-producing *E. coli* isolates [52]. A study in a Swedish university hospital conducted by Giske et al. reported 637–830 ESBL-producing *E. coli* isolates per year, with overall resistance to mecillinam of 5%–6% [38]. A German study investigating resistance to mecillinam in *E. coli* isolates from urine specimens of adult primary care patients in Germany in 2019/2020 reported that 42 of 46 (91.3%) ESBL-producing isolates (80.4% of samples from female patients) were inhibited by mecillinam [30]. In an Australian study of urinary specimens with microscopy consistent with UTI, mecillinam susceptibility of *E. coli* resistant to third-generation cephalosporin was actually marginally higher than that reported for *E. coli* susceptible to third-generation cephalosporin (87.8% [86/98] vs 83.2% [84/101], respectively) [31].

The comparative susceptibility of mecillinam was evaluated in 394 multidrug-resistant (MDR) Enterobacterales species, with different resistance mechanisms, isolated (in 2019 and 2020) from urinary specimens with elevated MICs of third-generation cephalosporins [49]. The most common species was *E. coli* (n = 198), and the most common resistance mechanism was ESBL (n = 273), followed by AmpC (n = 132) and carbapenemases (n = 12). Of the 198 MDR *E. coli*, mecillinam showed 97% susceptibility to the 177 ESBL strains [49].

### Susceptibility of Carbapenem-Resistant *E. coli* to Mecillinam

In a German study investigating the activity of several antibiotics against carbapenem-nonsusceptible Enterobacterales without carbapenemase production (isolates identified from a national reference library), mecillinam was active against 65% of *E. coli* isolates [61]. Another German study reported a lower susceptibility rate, using samples from a collection of molecularly characterized carbapenemase-producing Enterobacterales from different clinical specimens; 40% of 30 carbapenemase-producing *E. coli* isolates were susceptible to mecillinam [60]. In the former study [61], resistance mechanisms included CTX-M, TEM-ESBL, and AmpC. In the latter [60], different mechanisms were at play, and the predominant carbapenemase in the 12 susceptible *E. coli* isolates was OXA-48 (n = 6).

A French study evaluated mecillinam susceptibility in a large collection of carbapenem-resistant Enterobacterales isolates (n = 8310) from the national reference center, including OXA-48-like and other carbapenemase producers [58]. Across 1943 carbapenem-resistant *E. coli* isolates, 84.2% were susceptible to mecillinam. In this *E. coli* group, susceptibility rates were 92.6%, 76.2%, 17.6%, and 0% for OXA-48-like, NDM, VIM, and KPC producers, respectively, and 75% for carbapenem-resistant non-carbapenemase-producing Enterobacterales (ESBL/AmpC) [58]. In a Greek study, 197 isolates containing OXA-48-like carbapenemase-producing Enterobacterales were identified from samples collected from individual patients in 9 tertiary care hospitals during 2012–2019, from clinical sites including the urinary tract, bloodstream, and central venous catheter [59]. In this study, 16 of the 17 *E. coli* isolates (94.1%) were susceptible to mecillinam.

### Susceptibility of Non–*E. coli* Uropathogens (Overall) to Mecillinam

Five studies reported the overall susceptibility of non–*E. coli* Enterobacterales to mecillinam [28, 45, 47, 48, 62] (Table 2). Across 3 of these studies, susceptibility to mecillinam ranged from 92.8% to 100% [47, 48, 62]. This included data from ECO-SENS, which reported resistance to mecillinam in 4.2%, 5.2%, and 1.6% of isolated *Proteus mirabilis*, *Klebsiella* spp., and other Enterobacterales species, respectively. The limited number of isolates of species other than *E. coli* did not permit country-specific analysis of these data [47]. A large UK study published more than 40 years ago investigated resistance to mecillinam in different settings, reporting resistance rates of <1% in general practice and antenatal settings, as well as among hospital outpatients; resistance was 3.1% among hospital inpatients [62]. A 2017/2018 study assessed the prevalence of antimicrobial resistance in Enterobacterales isolates recovered from urinary tract samples in France, including 134 162 Enterobacterales isolates, of which 28% were non–*E. coli* species [28]. Mecillinam had the highest susceptibility to *Citrobacter koseri* (97.9%), with several other species also showing low resistance to mecillinam. The lowest susceptibility rates were seen for mecillinam in *Serratia marcescens* (30.8%) and *Morganella morganii* (29.5%).

### Susceptibility of ESBL- or AmpC-Producing Phenotypes of Non–*E. coli* Uropathogens to Mecillinam

A total of 10 studies reported susceptibility data for resistant phenotypes of non–*E. coli* uropathogens [49, 50, 52, 53, 58–61, 63, 64] (Table 2).

UK studies have reported that mecillinam susceptibility among ESBL-producing Enterobacterales ranged from 83% to 95%, using samples from a range of clinical settings (eg, community and hospital, male and female) [52, 63, 64]. Data from clinical samples (including some bloodstream infections) collected in France indicate that the susceptibility of ESBL-producing *Klebsiella pneumoniae* (*K. pneumoniae*) to mecillinam is ∼90% [53]. A German analysis of 394 MDR Enterobacterales included *Klebsiella* spp. (n = 87), *Enterobacter* spp. (n = 52), *Citrobacter* spp. (n = 34), and others [49]. Mecillinam susceptibility rates were 96% for *Enterobacter* spp., where the predominant resistance mechanism was AmpC. Susceptibility rates were between 69% and 76% for *Klebsiella* spp. (resistance mechanism not specified), *Citrobacter freundii* (largely AmpC), and *K. pneumoniae* (>90% ESBL), while *M. morganii* (>90% AmpC) was completely resistant to mecillinam [49].

### Susceptibility of Carbapenem-Resistant Non–*E. coli* Enterobacterales to Mecillinam

Five studies provided data for non–*E. coli* carbapenem-resistant Enterobacterales [49, 58–61] (Table 2). As with the carbapenem-resistant *E. coli* data, a much greater degree of heterogeneity was apparent in susceptibility rates, with variability seen across strains, carbapenemase type, and geography.

Reported susceptibility rates for carbapenem-resistant or carbapenemase-producing *K. pneumoniae* varied from a low of 8.5% in 1 study [60] to a high of 66.9% in another [58]. The latter study examined different mechanisms of carbapenemase-related resistance, showing that mecillinam susceptibility for *K. pneumoniae* was highest with OXA-48-like (84.2%) or NDM (72.1%) resistance mechanisms and lowest where the carbapenemase was VIM or KPC (16.2% or 0%, respectively) [58]. Another French study also reported 0% susceptibility of mecillinam to VIM, NDM, and KPC strains, although it should be noted that the numbers of isolates were <10 [49]. Levels of susceptibility to mecillinam in carbapenem-resistant *Enterobacter cloacae* in several studies ranged from 53.8% to 80% [58, 60, 61].

### Susceptibility to Mecillinam in the United States

To support the clinical development of pivmecillinam in the United States for the treatment of uUTI, the activity of mecillinam has been compared with other antibiotics against Enterobacterales isolates from patients with UTI in the United States during 2018 and 2019 [65, 66]. A total of 1090 isolates were tested for the 2018 analysis, and 1075 were tested for the 2019 analysis. The activity of the antibiotics was tested by CLSI methodology, and susceptibility was interpreted according to CLSI guidelines. In the 2018 analysis, mecillinam MIC_50_ and MIC_90_ were 0.25 µg/mL and 4 µg/mL, respectively, and 94.5% of isolates were susceptible [65]. In the 2019 analysis, presence of ESBLs were found in 9.6% of *E. coli* and 50% of *K. pneumoniae* isolates [66]. Susceptibility to mecillinam was 95%, and MIC_50_ and MIC_90_ values for mecillinam were 0.25 μg/mL and 2 μg/mL, respectively [66]. In this same 2019 analysis, 22.6% and 26.2% of isolates were nonsusceptible to ciprofloxacin and TMP-SMX, respectively. Among the ciprofloxacin-nonsusceptible isolates, 91.4% were susceptible to mecillinam, and the MIC_50_ and MIC_90_ values for mecillinam were 0.5 and 8 mg/L, respectively. Among the TMP-SMX-nonsusceptible isolates, 93.6% were susceptible to mecillinam, and the MIC_50_ and MIC_90_ values for mecillinam were 1 and 4 mg/L, respectively [66].

### The Role of Pivmecillinam in the Management of uUTIs: Concluding Remarks

This systematic review shows that mecillinam, historically and currently, demonstrates a good level of activity against *E. coli* and several other Enterobacterales that cause uUTIs—even those with resistance to other antimicrobials. Across 21 published studies, the susceptibility of mecillinam to *E. coli* exceeded 83% in each of those studies, exceeded 90% in 17 of the studies, and exceeded 95% in 15 of the studies (Table 1).

Susceptibility rates of mecillinam for ESBL *E. coli* range from 82.5% to 97.9% (Table 1). Further, we show that mecillinam has good activity in non–*E. coli* Enterobacterales, with susceptibility rates frequently in the region of ≥90% (Table 2). For example, the susceptibility of mecillinam to ESBL-producing *K. pneumoniae* (n = 50 isolates) was 90% in a 2018 French study [53]. While most studies observed pivmecillinam susceptibility rates in excess of 80% against ESBL-producing *E. coli* and other Enterobacterales [29–31, 34, 38, 42, 49–57, 63, 64], the microbiological activity of pivmecillinam against carbapenem-resistant Enterobacterales (CRE) was found to be highly variable across strains, carbapenemase types, and geographic regions. Available data suggest that the microbiological activity of pivmecillinam against CRE at its current susceptibility breakpoint is largely restricted to isolates producing OXA-48-like carbapenemases and some NDM-1 carbapenemases and that pivmecillinam activity against CRE is more frequent in *E. coli* and *E. cloacae* than in other species [58–61]. Pivmecillinam has limited microbiological activity at its current breakpoint against CRE that produce KPC or VIM [58]. While the microbiological data presented in this paper suggest that pivmecillinam may be an option for some CRE uUTIs if the pathogen is reported to be susceptible to mecillinam, there are currently no preclinical pharmacokinetic/pharmacodynamic data and limited clinical data to indicate that pivmecillinam is effective for patients with uUTIs caused by CRE. Further research is needed to determine if pivmecillinam can be used for CRE uUTIs when they are determined to be susceptible to mecillinam.

Recommendations for the choice of antimicrobial for treating uUTIs have needed to evolve in recognition of increasing numbers of resistant microbial strains. Notably, resistance to both fluoroquinolones and TMP-SMX, 2 of the most used therapeutic agents in adult patients with uUTIs, exceeds 20% in most US regions among adult patients with uUTIs due to *E. coli* [6, 8, 67]. With limited numbers of novel antimicrobials entering clinical practice for uUTI, there is attention on revival of older agents that could be used more widely [38]. Pivmecillinam, the oral prodrug of mecillinam, has been successfully used to treat uUTI in Europe and Canada for decades and is well placed to be considered as one of the empiric agents in adult patients with a suspected or documented uUTI due to Enterobacterales [22, 38]. Clinical practice guidelines from the IDSA recommend pivmecillinam as a first-line antibiotic treatment option, and in 2024 pivmecillinam received US FDA approval for the treatment of uUTI (185 mg orally 3 times daily for 3 to 7 days as clinically indicated) [14, 23]. Short (3-day) courses of pivmecillinam have demonstrated high levels of clinical success and may have the potential to reduce management costs of uUTIs [21]. An analysis using US-focused conceptual health care decision-analytic modeling has suggested that pivmecillinam has the potential to reduce the economic burden associated with inappropriate treatment of adult outpatients with uUTIs, especially in patients at high risk for resistance [7].

Several things should be noted when interpretating the findings of this literature review. National surveillance programs that examine susceptibility of key antibiotics annually are established in some countries, for example, in Norway, Sweden, and Denmark, as described in a recent review [22]. However, this is not the case for all countries, and the current review is limited to available published data. That said, we did not observe any evidence or trend toward changes in resistance to mecillinam at different time periods, as is well documented with other first-line uUTI agents with high usage [9]. It is important to acknowledge that all susceptibility data described herein are based on breakpoints of 8 mg/L and that these findings may need to be revisited if recommended breakpoints change in the future.

While not the focus of this review, more data are needed to fully understand the activity of pivmecillinam against Enterobacterales that are resistant to fluoroquinolones or TMP-SMX. In a 2019 US surveillance study of uropathogens recovered from patients with uUTIs, susceptibility to mecillinam among the ciprofloxacin and TMP-SMX-nonsusceptible isolates was 91.4% and 93.6%, respectively [66]. In view of the widespread use of and increasing resistance toward fluoroquinolones and TMP-SMX in the treatment of uUTI, further research would be valuable to determine the utility of pivmecillinam in patients with uUTIs caused by Enterobacterales that are resistant to fluoroquinolones or TMP-SMX. Finally, clinical and microbiological success rates were not examined within this review, the scope of which was limited to examination of antibiotic resistance as determined by currently approved AST methodology, and susceptibility is not necessarily a direct correlate of clinical success.

In conclusion, data from this literature review suggest that pivmecillinam has high microbiological activity at its current susceptibility breakpoints against *E. coli* and other Enterobacterales, including those that produce ESBLs. Importantly, there is no clear evidence to suggest that resistance to mecillinam among common uropathogens has increased during the >40 years of its use in practice. There is a wealth of existing clinical experience available for pivmecillinam, particularly from Nordic countries. In these regions, pivmecillinam use is reserved for uUTIs, and adoption of pivmecillinam as the standard treatment option for the majority of uUTI cases has spared the use of other antibiotic agents such as quinolones, co-trimoxazole, and amoxicillin-clavulanic acid for this highly prevalent condition. These considerations would suggest that pivmecillinam is an appropriate first-line treatment option for patients with uUTI in the current prescribing climate.
